# Modeling zoonotic cutaneous leishmaniasis incidence in central Tunisia from 2009-2015: Forecasting models using climate variables as predictors

**DOI:** 10.1371/journal.pntd.0005844

**Published:** 2017-08-25

**Authors:** Khouloud Talmoudi, Hedia Bellali, Nissaf Ben-Alaya, Marc Saez, Dhafer Malouche, Mohamed Kouni Chahed

**Affiliations:** 1 National Engineering School of Tunis, ENIT, Tunis El Manar University, Tunis, Tunisia; 2 Research Unit on Modeling, Statistics and Economic Analysis (MASE, ESSAI), High School of Statistics and Information Analysis (ESSAI), University of Carthage, Tunis, Tunisia; 3 Department of Epidemiology and Statistics, Abderrahman Mami Hospital, Ariana, Tunisia; 4 Research Unit "Analysis of the Effects of Environmental and Climate Change on Health", Department of Epidemiology and Statistics, Abderrahmen Mami Hospital, Ariana, Tunisia; 5 Department of Epidemiology and Public Health, Faculty of Medicine of Tunis, Tunis El Manar University, Tunis, Tunisia; 6 National Observatory of New and Emergent Diseases, Tunis, Tunisia; 7 Research Group on Statistics, Econometrics and Health (GRECS), University of Girona, Girona, Spain; University of Minnesota, UNITED STATES

## Abstract

Transmission of zoonotic cutaneous leishmaniasis (ZCL) depends on the presence, density and distribution of *Leishmania major* rodent reservoir and the development of these rodents is known to have a significant dependence on environmental and climate factors. ZCL in Tunisia is one of the most common forms of leishmaniasis. The aim of this paper was to build a regression model of ZCL cases to identify the relationship between ZCL occurrence and possible risk factors, and to develop a predicting model for ZCL's control and prevention purposes. Monthly reported ZCL cases, environmental and bioclimatic data were collected over 6 years (2009–2015). Three rural areas in the governorate of Sidi Bouzid were selected as the study area. Cross-correlation analysis was used to identify the relevant lagged effects of possible risk factors, associated with ZCL cases. Non-parametric modeling techniques known as generalized additive model (GAM) and generalized additive mixed models (GAMM) were applied in this work. These techniques have the ability to approximate the relationship between the predictors (inputs) and the response variable (output), and express the relationship mathematically. The goodness-of-fit of the constructed model was determined by Generalized cross-validation (GCV) score and residual test. There were a total of 1019 notified ZCL cases from July 2009 to June 2015. The results showed seasonal distribution of reported ZCL cases from August to January. The model highlighted that rodent density, average temperature, cumulative rainfall and average relative humidity, with different time lags, all play role in sustaining and increasing the ZCL incidence. The GAMM model could be applied to predict the occurrence of ZCL in central Tunisia and could help for the establishment of an early warning system to control and prevent ZCL in central Tunisia.

## Author summary

Zoonotic cutaneous leishmaniasis is a human vector-borne disease caused by the parasite *Leishmania major* and is well spread in rural areas where human resources in public health and infrastructure are limited. The cycle of transmission of the disease is complex because of the impact of climate change. In this study we evaluated the impact of bioclimatic factors on the transmission of the disease in three districts of Sidi Bouzid, central Tunisia. We found that the occurrence of zoonotic cutaneous leishmaniasis is mainly related to average temperature with 4 months lags, rodents' density lagged 2 months, relative humidity with 4 months lags and cumulative rainfall lagged 1 month. We also confirmed that our best-fit model predict well the occurrence of the disease.

## Introduction

Cutaneous leishmaniasis (CL) is a neglected tropical disease widespread in the Middle East, the Mediterranean basin and North Africa [1,2]. Most of the cases occur in the arid and semi-arid regions [3]. The circumstances of the dynamic leishmaniasis disease are often complex and varying according to environmental, demographic and human behavioral factors [4]. For cutaneous leishmaniasis, the parasite is transmitted by infected female sand fly vectors. Meanwhile rodents serve as the reservoir hosts [5–7]. The clinical form of cutaneous leishmaniasis occurring in humans, causes skin lesions and permanent scars, mainly on the face, arms and legs.

In Tunisia, the incidence of cutaneous leishmaniasis climbed from only one endemic governorate in 1983 to 15 endemic governorates in 2014 [8]. In fact, data from 10 governorates show that in 2014, 23% of the population is at risk. While a total of 57 591 cases were reported during the period from 1998 to 2007 [8].

Three clinical-epidemiological forms of cutaneous leishmaniasis identified in Tunisia, vary across regions [9]. In the North of Tunisia, Sparodic Cutaneous Leishmaniasis (SCL), one of the three forms of CL, is induced by *Leishmania infantum* MON-24. In central Tunisia, ZCL caused by *Leishmania major* MON-25 is considered dominant and distributed across arid zones [10]. The third form of CL: Chronic Cutaneous Leishmaniasis (CCL), generated by *Leishmania killicki* MON-8, was detected in the South-East of Tunisia [11]. According to Kallel et al. [12], the original foci were spread (for ZCL from the Center to the North and the South; for CCL from the South-East to the North; and for SCL from the North to the Center of the country).

ZCL remains primarily a disease responsible for considerable morbidity and disfigurement [2]. The disease is endemic, essentially in the rural areas of southern and central regions of Tunisia where infrastructure is limited and sanitation is inadequate [13–15]. In these regions, the climate is favorable to the development of sand fly species, and consequently to the transmission of the disease; so the population is exposed while managing farm activities [16].

However, the association between climate factors and ZCL incidence was not clearly elucidated. The way climate factors influence transmission of the disease became the main research concern. In fact, recent studies mainly used time series analysis to assess the relationship between climatic (or environmental) factors and daily (or monthly) records of the disease. Diverse models were used, and the choice of a model may have large influence on quantifying climate effects. The most used model of time series analysis in the field of epidemiology, is the Autoregressive Integrated Moving Average (ARIMA) analysis [17,18]. This model consider the order of data points and adjacent points in time [19]; which allows an estimation of autocorrelation and trend. These models adjust for variation due to previous observations, trend over time and variation in the observation that cannot be predicted from previous observations [20,21].

Although ARIMA process can be a robust technique for improving the quality of prediction, it assesses linearly the relation between the response variable and the predictor covariates. However, recent studies have identified non-linear relations of climate factors on ZCL incidence. Failure to model relations correctly can lead to model misspecification that can affect the error structure of the model. Studying non-linear time series analysis is still limited compared to linear series.

Generalized additive models (GAM) [22] have been used in environmental epidemiology [23–26]. GAMs are semi-parametric regression methods that relate the response variable to smoothed functions of potential explanatory variables via a link function [22]. Unlike parametric methods that impose the form of the trend to be used in the model, GAMs allow data to decide about this trend. In environmental epidemiological studies, the response variable may also be correlated. It is necessary to embody autocorrelation of the dependent variable when modeling. However, few works were interested in nonparametric regression when correlated observations are detected [27].

In this paper, we first aimed to model the relationship between the incidence of ZCL with the underlying predictor factors using the generalized additive models and its extension, the generalized additive mixed models considering the autocorrelation; and second, to predict the occurrence of the disease based on the best-fit model.

## Materials and methods

### Ethics statement

The whole project was approved by the ethical committee of Pasteur Institute in Tunis, Tunisia. But in this paper we presented the results of an ecological study with monthly number of ZCL cases and climate variables.

### Study area

**Fig 1:**
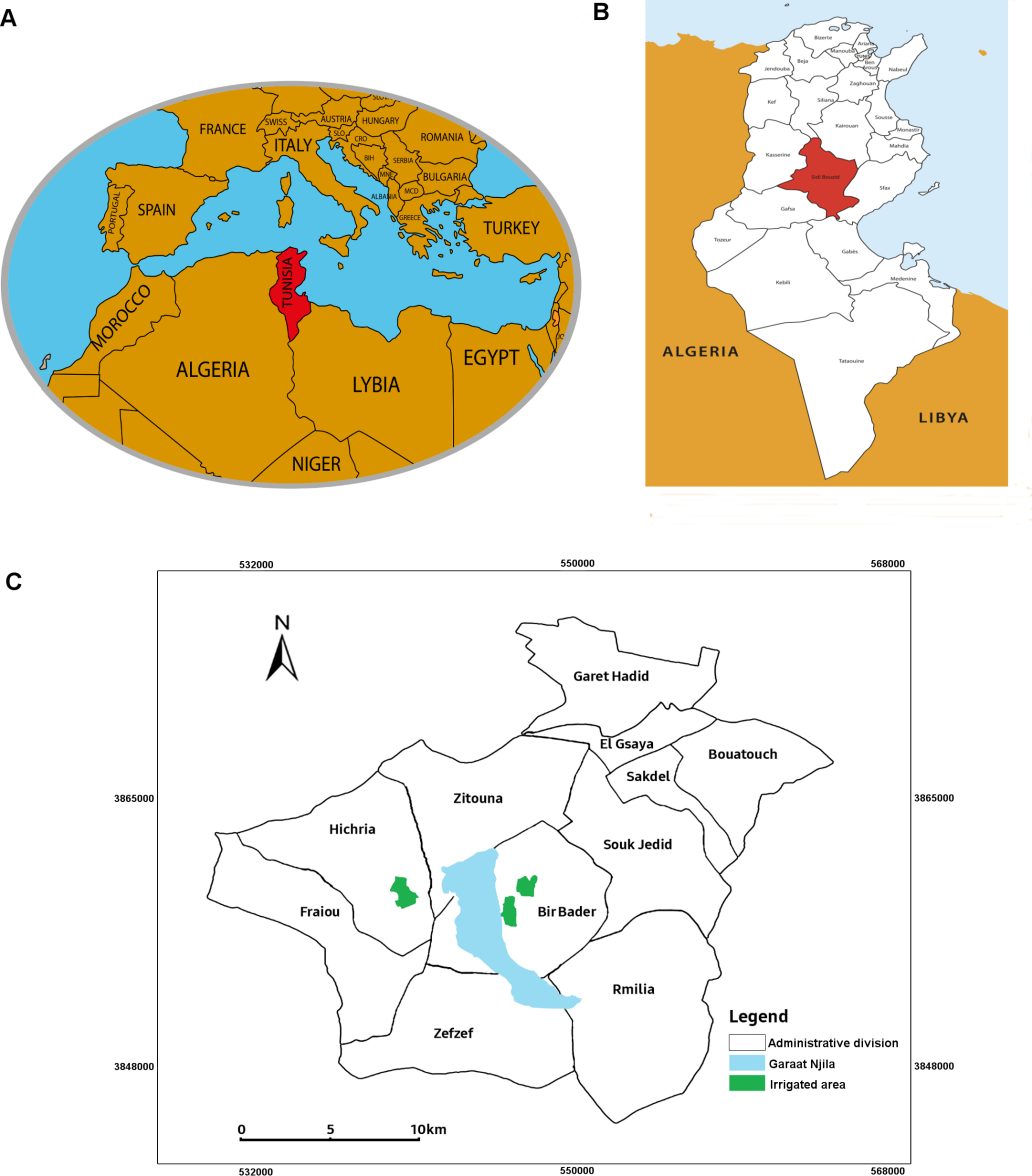
Spatial distribution of dwellings included in the study. (A) Location of Tunisia within the Mediterranean basin. (B) Location of Sidi Bouzid governorate within Tunisia. (C) Location of the study areas.

Our study area covers three districts of Bir Badr, Hichria and Zefzef. These districts are selected all around the salt pan "Garaat Njila" located in the governorate of Sidi Bouzid, central Tunisia (Fig 1). Such areas are characterized by a semi-arid climate and a long-lasting emergency of the disease.

### ZCL data

In our work monthly ZCL records were used, from July 2009 to June 2015. Data were obtained from an active system of epidemiologic surveillance, implemented in Sidi Bouzid, central Tunisia. All new cases of people seeking treatment at primary health care facilities and other cases notified among patients' neighbors and families by the active research of the nursing stuff were included in this surveillance. Also, all schools in this area have been asked to check and notify all ZCL cases among students. Moreover, members of the research team performed a community-based active ZCL surveillance, and notified cases in schools with a clinical suggestive form of cutaneous leishmaniasis were diagnosed by physicians and nurses from the health care facilities. Parasitologic diagnosis of ZCL lesions was carried out only for a group of patients using direct examination, skin culture, PCR TagMan and PCR high-resolution melting. No laboratory exam was undertaken by dint of the good knowledge of the disease by the medical staff, the population in this region, the high sensitivity and specificity of clinical diagnosis.

The number of monthly ZCL notifications was accounted according to the date of the lesion onset for the period between July 2009 and June 2015 after being reported on a standardized sheet. Data were anonymized and for this study, we only count the number of cases monthly. There is no information about patients.

The whole project was approved by the ethical committee of Pasteur Institute in Tunis, Tunisia, but in this paper we presented the results of an ecological study with monthly number of ZCL cases and climate variables.

We used 2009–2014 counts for the development of the predictive models and set aside 2014–2015 counts for independent validation of the prediction.

### Environmental and bioclimatic variables

The bioclimatic variables used for this study were monthly data between July 2009 and June 2015, recorded from a private station implemented in the study area. The variables collected were: average minimal temperature, average maximal temperature, average of averages temperatures, cumulative precipitation, average relative humidity, average wind speed, maximum wind speed, and average rodent density estimated according to their activity.

### Statistical analysis

Our study used real data of ZCL notifications, environmental and bioclimatic variables from July 2009 to June 2015. We used a generalized additive model (GAM) and a generalized additive mixed model (GAMM) with natural cubic splines. These models were used to assess the relation between ZCL incidence and climate factors (temperature, rainfall, relative humidity, wind speed and rodents' density). These covariates were included into the model as fixed effects. However, autoregressive terms were considered as random effects.

#### Cross-correlation analysis

The first stage of the analysis was to determine the optimal lags of explanatory covariates to be used in the models. Therefore, we used cross-correlation function (CCF). The latter allowed us to determine the significant delayed dependencies, among various lags, in the association between explanatory variables and ZCL cases [28].

The cross-correlation terms are retained in the model if the absolute value of cross-correlation coefficient is two times larger than the standard error [29].

#### Generalized additive model (GAM)

The different patterns of associations between environmental and bioclimatic variables defined by temperature, rainfall, relative humidity, wind speed and rodents' density, with ZCL cases were assessed using Quasi-Poisson generalized additive model (GAM) [30–31], using natural cubic splines. We used the "mgcv" package version 1.8–7 designed by S. Wood [30] on the R statistical software (version 3.1.2) [32].

A GAM is a semi-parametric extension of the generalized linear model (GLM) where the linear predictor ∑β_j_X_j_ is replaced by a sum of smooth functions of the covariates ∑f_j_(X_j_) [22]. Both GLM and GAM allow the exploration of nonlinear data structures in the context of exponential family distributions (e.g. Quasi-Poisson and Negative Binomial), and use link functions to establish relationships between the mean of the outcome variable and the predictors [33,34]. Unlike GLMs in which the researcher have to impose the form of the trend existing in the data, GAMs automatically identify and estimate the optimal degree of nonlinearity of the model directly from the data [30]. The general structure of GAM can be written as:

$$g(E(y_{t}))=g(\mu_{t})=X_{i}^{*}\theta +f_{1}(x_{1t})+f_{2}(x_{2t})+f_{3}(x_{3t},x_{4t})+\cdots +\epsilon_{t}$$

where g is a link function, *μ*_*t*_ is the expectation of the response variable, y_t_, $X_{i}^{*}$ is a parametric model component, *θ* is the corresponding parameter vector and *f*(.) are smoothing functions of predictor variables, *x*_*t*_, estimated according to data. These smooth functions make the residual sum of squares (RSS) small and smooth to avoid the problem of overfitting the data. Thus, the function *f* minimizes:

$$\sum_{i=1}^{n}{\left(y_{i}-f(x_{i})\right)}^{2}+\lambda \int {f"(t)}^{2}dt$$

where *λ* is a non-negative tuning parameter. The term $\sum_{i=1}^{n}{\left(y_{i}-f(x_{i})\right)}^{2}$ is a function that encourages *f* to fit the data well, and the term *λ* ∫ *f*"(*t*)^2^*dt* is a penalty term that adjust too "wiggliness" in the smoothing function, to avoid overfitting curves to the data. It encourages *f* to be smooth. The larger the value of *λ*, the smoother *f* will be. The tuning parameter *λ* controls the roughness of the smoothing functions. This parameter is determined in such a way that the generalized cross-validation is as small as possible [35].

Several GAMs were employed and generated to best fit the data. We started by performing a complete model, including all independent variables and then we proceeded to variable selection based on a stepwise backward process, using the Generalized Cross-Validation (GCV) score [30] as selection criteria. The best parsimonious model was selected based on the lowest GCV. Autocorrelation of model residuals was later checked using autocorrelation and partial autocorrelation function plots. According to Wood [36], the significance of each smooth term in the model was checked using Bayesian confidence limits for the smooths.

#### Generalized additive mixed model (GAMM)

GAMM is the extension of GAM, which is proposed to overcome overdispersion and autocorrelation in observations. Compared to GAM, this class of models allows flexible dependence of a response variable on independent variables, using nonparametric regression. Also, it includes correlation between observations in the model by using random effects [37]. Suppose the outcome variable, *y*_*i*_, and *p* covariates, *x*_*i*_ = (1,*x*_*i*1_,…,*x*_*ip*_)^*T*^, associated with fixed effects and a *q* × 1 vector of covariates *z*_*i*_ associated with random effects. Given a *q* × 1 vector *b* of random effects, the observations *y*_*i*_ are assumed to be conditionally independent with means $E(y_{i}|b)=y_{i}^{b}$ and variances $var(y_{i}|b)=\phi m_{i}^{-1}\upsilon (y_{i}^{b})$ where *υ*(.) is a specified variance function, *m*_*i*_ is a prior weight and *ϕ* is a scale parameter, and follow a generalized additive mixed model [37] written as:

$$g(\mu_{i}^{b})=\beta_{0}+f_{1}(x_{i1})+\cdots +f_{p}(x_{ip})+z_{i}^{T}b$$

where *g*(.) is a link function, *f*_*j*_(.) are smooth functions, the random effect *b* is assumed to be distributed as N(0,D(θ) and *θ* is a vector of variance components.

The main property of the GAMM model is that independent factors are used in additive nonparametric functions in the fixed effect, and correlation between observations are considered in the random effect.

Independent validation of the best-fit models was performed using data from July 2014 to June 2015. Agreement between model predictions of ZCL cases using the best-fit model, and the actually reported ZCL cases during 2014–2015, was evaluated through confidence interval analyses.

## Results

### Explanatory analysis

**Fig 2:**
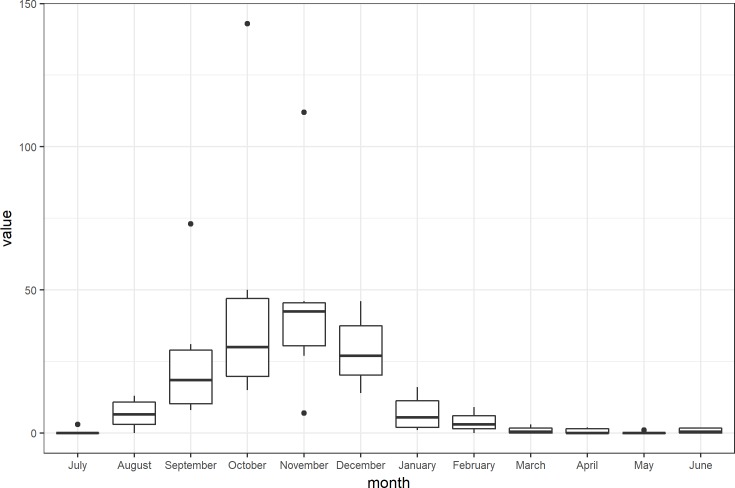
Box plot with monthly ZCL incidence. Data was monthly aggregated from July 2009 to June 2014.

**Fig 3:**
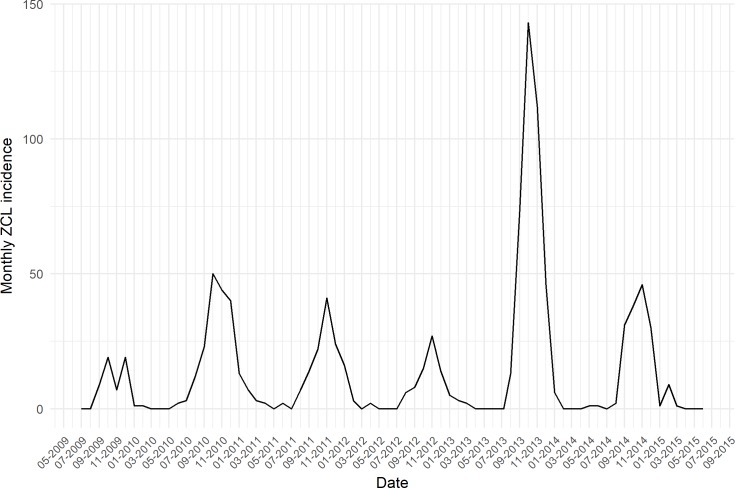
Month and year of ZCL lesion onset.

There were 861 notified ZCL cases over the study period, from July 2009 to June 2014 in the three districts of Sidi Bouzid, central Tunisia. The peak of ZCL mainly occurred from August to December during the same epidemiological year (Fig 2). Also, this result is stressed by the seasonality test which rejected the equality of months (Kruskal-Wallis = 46.57, df = 11, p < 0.001). In Fig 3, a temporal variation of the ZCL incidence, is revealed during the whole study period, with an outbreak of 143 cases in October 2013, during the fifth epidemic season. Large values on the incidence of the disease were also seen during the second and the third epidemic seasons, but very low values were recorded in the first and fourth epidemic seasons.

### Relationship between climatic variables and ZCL incidence

Cross-correlation analysis (Table 1) showed that several bioclimatic parameters were significantly associated with ZCL cases at various monthly lags. The most significant associations (within all environmental and bioclimatic variables) were for average temperature (Tavg) at 2-, 3- and 4-months lag, cumulative rainfall (rainf) lagged one month, relative humidity (relative_humidity) delayed 4 months, and rodent's density (rodens) at 2-months lag.

**Table 1:** Cross-correlation coefficients of bioclimatic and environmental variables with the ZCL cases.

Lag(months)	Humid(%)	Rainf(mm)	Tavg(°C)	Tmin(°C)	Tmax(°C)	Rodensiy	Aws	Mws
**0**	0.199	0.044	-0.038	0.024	-0.113	-0.278*	-0.180	-0.241
**-1**	0.210	0.274*	0.204	0.203	0.136	0.016	0.165	-0.195
**-2**	-0.050	0.036	0.275*	0.168	0.188	0.267*	-0.159	-0.050
**-3**	-0.257	0.062	0.390*	0.220	0.274*	0.026	-0.183	-0.045
**-4**	-0.358*	-0.197	0.255*	0.129	0.241*	-0.033	0.092	0.178
**-5**	-0.178	-0.040	0.130	-0.009	0.123	-0.061	0.159	0.060
**-6**	0.019	0.026	0.089	-0.002	-0.014	-0.017	0.259*	0.178
**-7**	-0.037	0.032	0.024	-0.050	-0.015	0.056	0.186	0.157
**-8**	0.041	0.031	-0.252*	-0.155	-0.140	0.070	0.145	0.182
**-9**	0.092	-0.015	-0.396*	-0.182	-0.259*	0.061	0.024	0.034
**-10**	0.024	-0.122	-0.376*	-0.161	-0.301*	-0.057	-0.080	-0.039
**-11**	0.190	-0.106	-0.296*	-0.137	-0.103	-0.069	-0.059	-0.081
**-12**	0.352*	0.029	-0.169	0.018	-0.132	-0.238	-0.193	-0.26*

* significant at 0.005 level.

The probability distribution of the ZCL incidence required for GAM model is slightly over-dispersed (Mean = 14.15; Standard deviation = 24.91). A Quasi-Poisson distribution fitted adequately the data.

We began with building GAM models to estimate the pattern of each influential variable on ZCL incidence. Then, we used the generalized cross validation (GCV) score to compare the statistical performances of different models. The lowest GCV value yields to the best fit model. Results from the best-fit GAM model with a Quasi-Poisson distribution showed a significant associations between ZCL incidence and accumulated rainfall lagged 1 month, average temperature lagged 4 months, relative humidity with 4 months lag and rodent's density lagged 2 months (lowest GCV = 2.23; deviance explained = 97.8%, dispersion parameter = 1.06 ≈ 1) (Table 2). So, the GAM model chosen to fit the data is given below:

$$E({ZCL}_{i})=\mu_{i}=\beta_{0}+f_{1}({month}_{i})+f_{2}({Tavg4}_{i})+f_{3}({relative\;humidity4}_{i})+f_{4}({pluv1}_{i})+f_{5}({rodens2}_{i})$$

where *ZCL*_*i*_ is the ZCL cases in the ith observation. The terms *f*_1_ to *f*_5_ are the smoothing functions, and *β*_0_ is the intercept (Table 2).

**Table 2:** Model estimates of the effects of environmental and bioclimatic variables on ZCL incidence.

Smooth terms	edf	F
**s(month)**	5.11	5.78***
**s(Tavg averaged over previous 4 months)**	7.44	6.72***
**s(rainfall averaged over previous 1 month)**	6.38	6.47***
**s(relative_humidity averaged over previous 4 months)**	3.10	1.90***
**s(avgrodens averaged over previous 2 months)**	4.24	5.71***
**Linear terms**	**Estimate**	**SE**
**Intercept**	1.15	0.12
**Explained deviance**	97.8%	
**GCV score**	2.23	

***Significant at the 0.000 level.

edf = effective degrees of freedom of the smooth function terms (edf > 1 indicate nonlinear relationships); F value is an approximate F-test, SE = asymptotic standard error.

**Fig 4:**
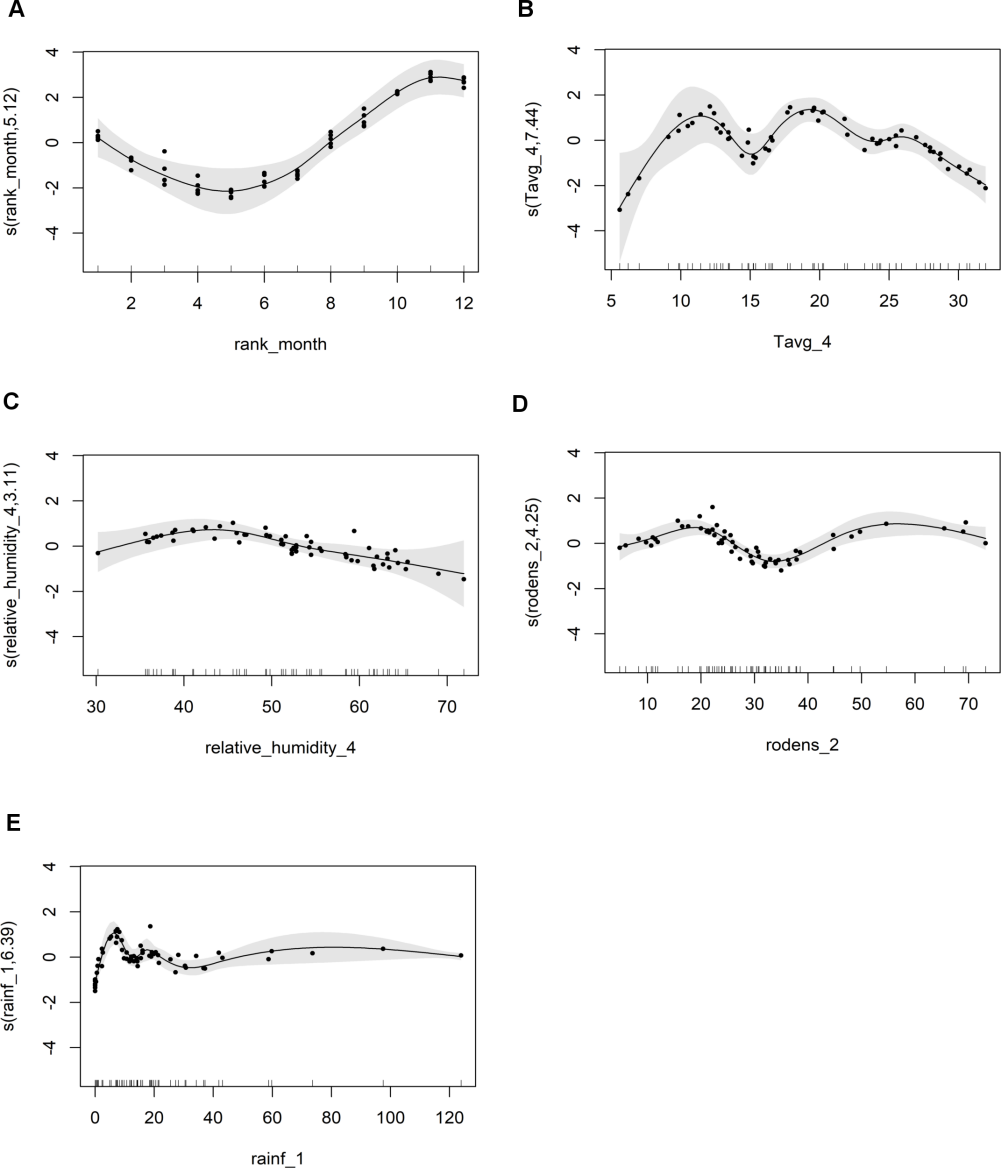
GAM-estimated relationships for months (A), temperature (B), relative humidity (C), rodent's density (D), and rainfall (E) on ZCL incidence. The x axis represents increasing variations in the bioclimatic covariates. The y axis indicates the contribution of the smoother to the fitted values.

The estimated effects of environmental and climate variables on ZCL incidence are shown in Fig 4, they revealed different patterns. All climate factors were statistically significant in a highly non-linear way. Fig 4A showed that during the same epidemiological season, months are characterized by a non-linear smooth with a positive effect for month 1 (January), a negative effect for months 2–7 (February to July), and an increase effect for months 8–12 (August to December). The month's period of negative effect coincides with the beginning of warm season which would be a favorable period for phlebotomies' activities. Average temperature delayed 4 months (Fig 4B) had a wiggly association with ZCL incidence (edf = 7.4). A positive effect was seen for temperatures ranging between 5°C—12°C and between 15°C—20°C. A negative effect was noted for temperatures ranging between 12°C—15°C and over 20°C. Relative humidity with 4 months lag had a non-linear association (edf = 4.3) with ZCL incidence (Fig 4C). An increase effect for relative humidity was seen from 30 to 45%, and a negative effect over 45%. The association between rodent's density 2-months lag and ZCL was wiggly (edf = 4.25) and is characterized by a general positive effect, except in the range of 20 to 30, where the effect is negative (Fig 4D). Monthly cumulative rainfall lagged 1 month had also a nonlinear association with ZCL incidence (edf = 6.3) and was found to have an increase effect reaching a peak at 10 mm (Fig 4E). Epidemic season was not significant in our model and had a non-significant linear effect on ZCL incidence. So, it was removed from the model.

**Fig 5:**
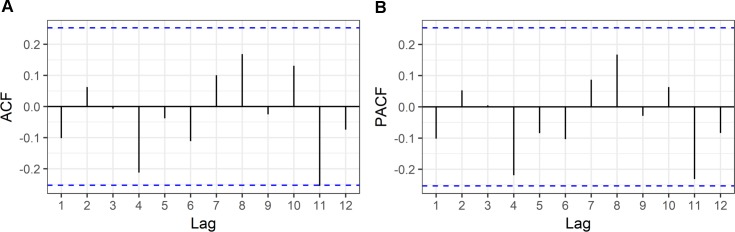
Autocorrelation and partial autocorrelation functions of the GAM model.

From Fig 5, the ACF and PACF plots of GAM model showed all lags fell within ±0.2 confidence bands implying that the GAM model might be an appropriate model. However, autocorrelated observations are not considered in this model. Here, the autocorrelation part was added to the best-fit GAM model as a random effect using the generalized additive mixed model.

**Fig 6:**
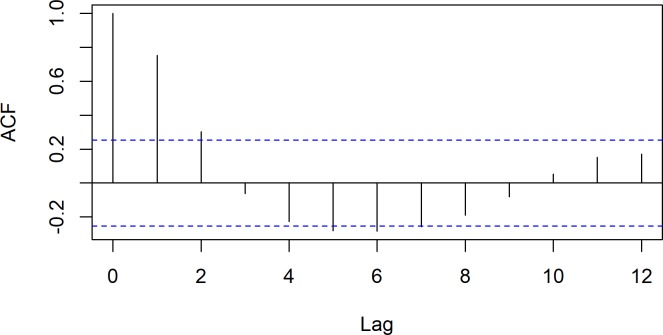
Autocorrelation function of ZCL incidence.

From the GAM model, the pattern of the relation between bioclimatic factors and ZCL incidence was assessed. However, the correlation between the observations of the response variable was not evaluated. So, the autocorrelation function of ZCL cases was checked (Fig 6), and revealed significant dependences.

**Fig 7:**
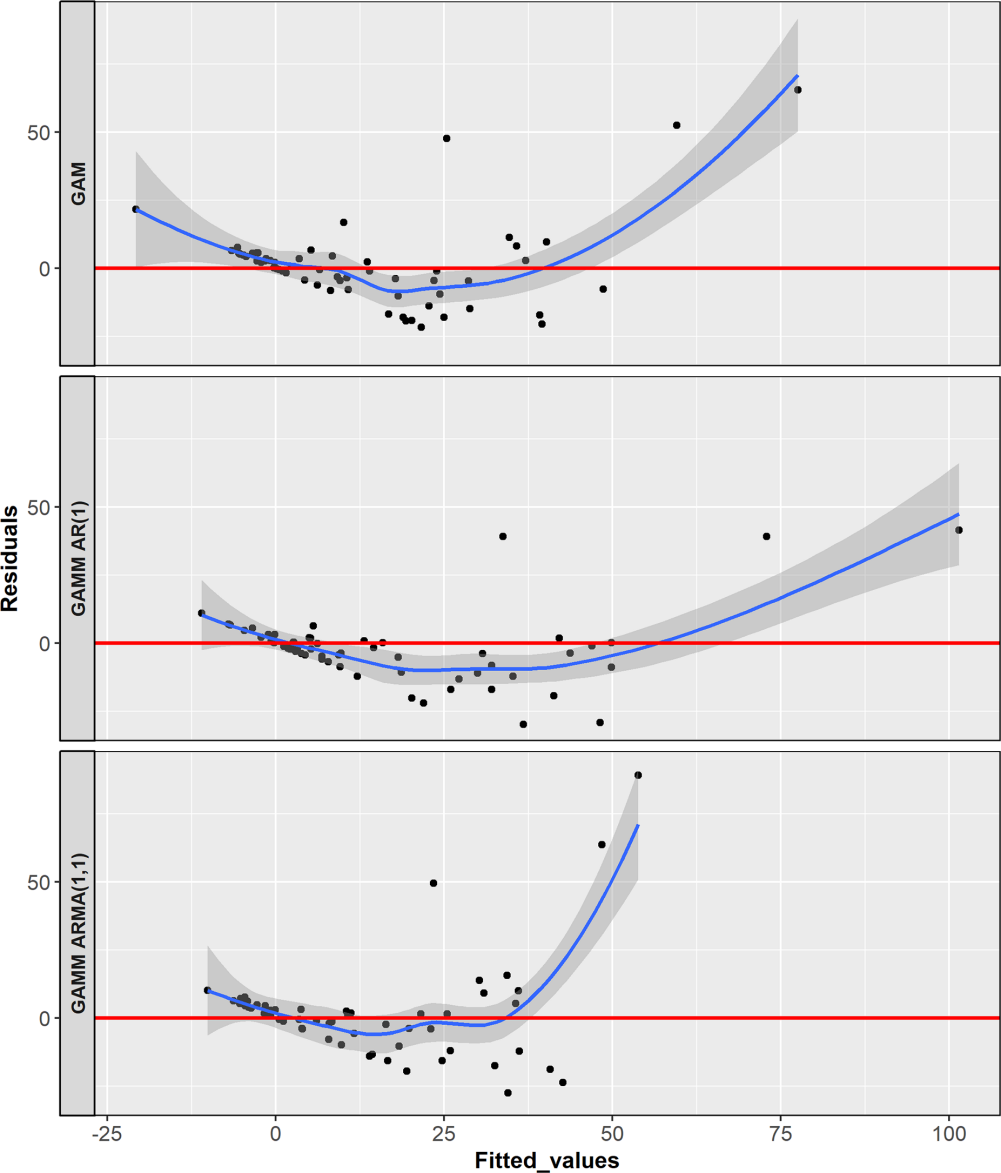
Comparison of residuals vs. fitted values from different GAM(M) models.

At this stage, we retained the significant variables from the best fit GAM model, and used its extension, GAMM, to consider the autocorrelation of observations and whiten the errors. So, we included Autoregressive Moving Average (ARMA) processes in the error structure. According to the autocorrelation function and the partial autocorrelation function plots of the dependent variable, the error structure might be an AR(1) or ARMA(1,1). Fig 7 showed that ARMA(1,1) process has residuals at low fitted values and could be retained as the best-fit GAMM model.

### Predicting ZCL cases

**Fig 8:**
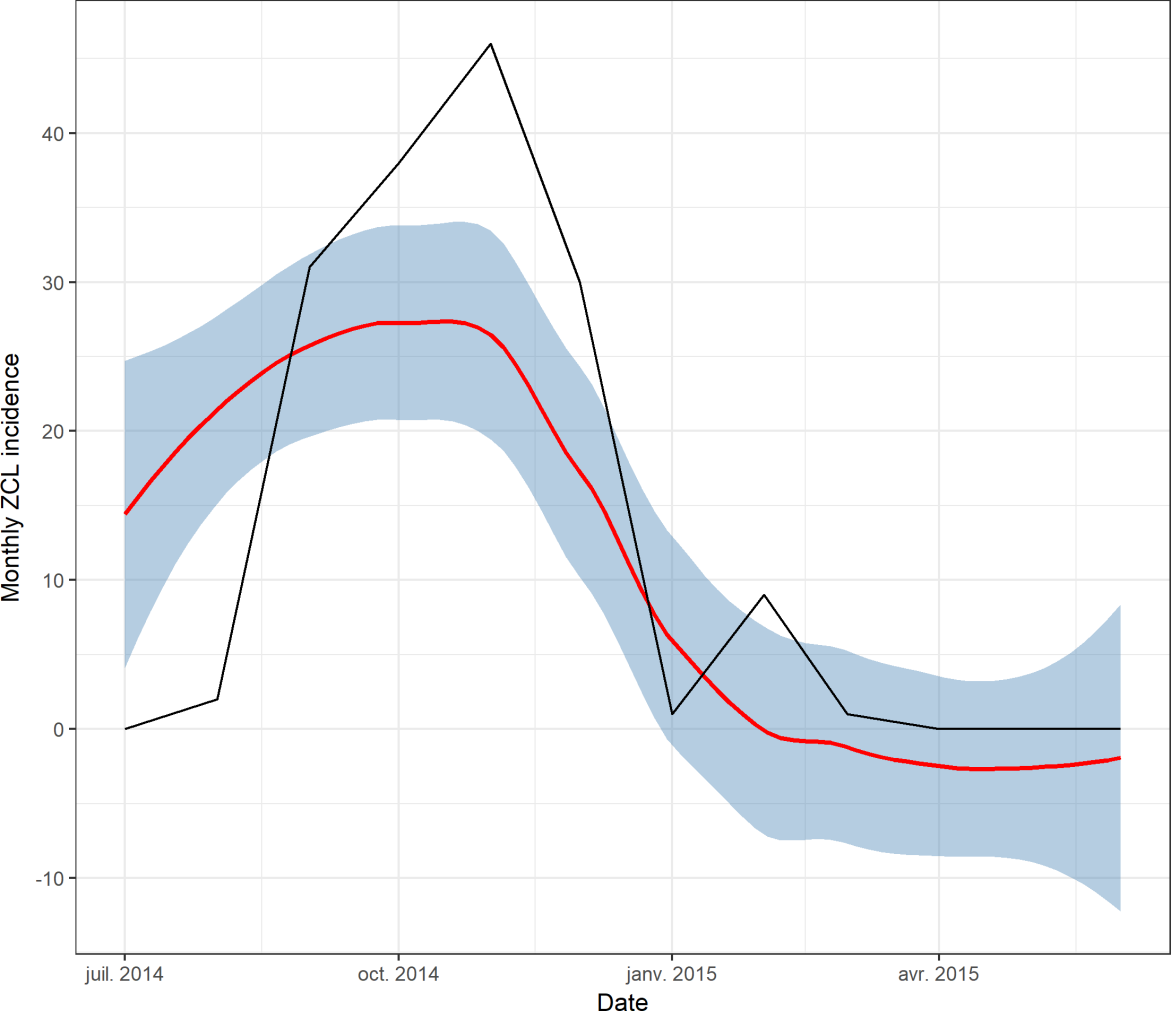
Predictive trend line from the final GAM with 95% predictive interval using data from July 2014 to June 2015.

The results of the prediction analysis, using the GAMM model and carried out using data from July 2014 to June 2015, were drawn with the original values (Fig 8). Results showed that prediction from GAMM approach gives a good prediction accuracy. The Pearson correlation value between predicted and original values of number of cases was 0.81 (IC = 0.46–0.94, p < 0.001). During the validation period, most monthly original values fell within the 95% confidence interval, presenting the obvious seasonal variation during months from September to December (Table 3).

**Table 3:** Prediction interval based on the final GAM model, with 95% predictive interval using data from July 2014 to June 2015.

Season	Month	Year	Original values	Predicted values	Prediction Interval
**6**	July	2014	0	19	[3.1; 34.8]
**6**	August	2014	2	14	[-1.2; 29.6]
**6**	September	2014	31	23	[8.3; 38.6]
**6**	October	2014	38	30	[14.1; 45.1]
**6**	November	2014	46	28	[11.9; 43.6]
**6**	December	2014	30	19	[0.9; 38.0]
**6**	January	2015	1	2	[-19.4; 23.2]
**6**	February	2015	9	0	[-19.2; 18.3]
**6**	March	2015	1	-2	[-21.4; 17.9]
**6**	April	2015	0	5	[-11.8; 21.0]
**6**	May	2015	0	-4	[-18.9; 10.4]
**6**	June	2015	0	-3	[-15.9; 10.1]

## Discussion

Although cutaneous leishmaniasis caused by *L*. *major* is considered as one of the most important diseases in Tunisia, few studies have been conducted studying the complicated relationships between the transmission of the disease and climatic and environmental variables. Previous studies conducted in Tunisia in 2000 and 2009 [38,39] revealed a significant relationship between Mediterranean visceral leishmaniasis and climatic factors. In such relationships, it is often very difficult to find a suitable mathematical function for fitting the relationship [40]. In 2012, Toumi et al. [41] used the autoregressive integrated moving average (ARIMA) models to demonstrate seasonality during the same epidemiologic year. They also applied Negative-Binomial generalized additive model (GAM) and generalized estimating equations (GEE) to quantify the relationship between temperature, rainfall, humidity and ZCL in central Tunisia. This study used monthly data from January 1991 to December 2007, it did not include wind speed and rodent's density in their GAM and GEE models. They reported that only humidity and rainfall lagged 12–14 months were significant predictors of ZCL cases in Sidi Bouzid, and that average temperature was not statistically a significant predictor of ZCL incidence [41].

In our study, we focused on the ZCL cases from July 2009 to June 2015 and we estimated lagged effects of diverse bioclimatic and environmental variables, including minimum temperature, maximum temperature, average temperature, cumulative rainfall, relative humidity, average wind speed, maximum wind speed, and rodent's density, on the incidence of ZCL using monthly data. We estimated significant effects of monthly average temperature, cumulative rainfall, relative humidity and rodent's density on ZCL incidence after accounting for distributed lag effects.

We found that cross-correlation between ZCL incidence and independent variables was not very high, although some were statistically significant. But, we retained the significant lagged variables in association with the incidence. In fact, the cycle of transmission of the disease is considered complex since the existence of three seasonality patterns: the first one stressed the importance of climate changes in the study region caused by the construction of dams and irrigation projects. Second, the climate variability may affect the density of rodents' reservoirs which is highly affected by the availability of chenopods, a plant exclusive food source of rodents, in the region [42]. So, transmission is better in warm season (May to September). The last seasonality is characterized by the onset of the disease and its development that occurs in cold season (October to May) [43].

Then, we identified the relationship between ZCL and the selected environmental and bioclimatic factors in Sidi Bouzid, central Tunisia. To explain this association, a Quasi-Poisson GAM regression was chosen to be the model adopted that integrates parametric and non-parametric terms. GAM is specifically designed to analyze data when the impact of the predictors on the outcome is not necessarily linear. The results of our research stated the robustness and flexibility of GAM to reveal meaningful curvatures in exploratory analyses and the good quality of fitting.

We found that the outbreak of ZCL was associated more with local environmental diversity than with climate factors. In fact, our results showed that rodent's density and weather variables could be used to predict ZCL transmission. To our best knowledge this is the first study incorporating rodent's density in the model, together with climate variables.

Our GAM model showed that the significant effect of ZCL incidence was associated with monthly cumulative rainfall lagged 1 month. In fact, rainfall would increase the density of the halophytic plant, chenopods, that constitute the food of rodent's reservoirs [6,7]. Consequently the reservoir density increases, and affects the ZCL transmission.

We also found that ZCL was associated with rodent density with 2 months lagged effect jointly with relative humidity lagged 4 months. Relative humidity has an influence on the survival of sandfly eggs and adults, the biting behavior of female adult sandfly, and laying of eggs. At the same temperature, egg hatchability of *Phlebotomus papatasi* increases as the relative humidity rises. The optimum relative humidity is 75% for saving eggs. When the humidity is too low, laying eggs will be affected, and adult mosquito mortality will increase. Increasing humidity will also facilitate feeding for the adult sandfly, enhancing its survival.

The results of our GAM model showed that average temperature lagged 4 months has significant effect on ZCL occurrence. According to some studies [44,45], temperature is one of the factors that determine the abundance of mosquitoes and the prevalence of mosquito-borne diseases.

Despite the flexibility of our model that provided a better assumption of the nature of relationships between each bioclimatic factors and the number of ZCL cases, one limitation has to be pointed out. The short-term time series used in this work may not validate our predictive model. However, we need to extend the number of observations up to 10 years to validate a robust predictive model. Such recommendations are essential to improve the model and may help governors to detect earlier an outbreak occurrence and reduce, as well as possible, number of ZCL cases.

A predictive model using spatiotemporal data needs to be the next goal to be accomplished in further studies towards the construction of an early warning system (EWS) of ZCL in Sidi Bouzid, central Tunisia. To this end a sustained surveillance and monitoring efforts of ZCL and climate factors are needed to provide time series sufficiently long for developing and evaluating forecasting models.

To conclude, a complex relationship between environmental, bioclimatic factors and ZCL occurrence was found in central Tunisia. Additive models offer flexible modeling tools for regression problems. Understanding the role of the environmental and bioclimatic factors in ZCL occurrence can help to guide government policy-makers towards the creation and implementation of more effective policies to tackle the disease, and has important implications for prevention measures.

## Supporting information

S1 Dataset (XLSX)
